# Insular cortex involvement in declarative memory deficits in patients with post-traumatic stress disorder

**DOI:** 10.1186/1471-244X-9-39

**Published:** 2009-06-18

**Authors:** Shulin Chen, Lingjiang Li, Baihua Xu, Jun Liu

**Affiliations:** 1 Department of Medical Psychology, the Seventh Hospital of Hangzhou, Zhejiang, PR China; 2 Department of Psychology, Zhejiang University, Hangzhou, Zhejiang, PR China; 3 Mental Health Institute, the Second Xiangya Hospital, Central South University, Changsha, Hunan, PR China

## Abstract

**Background:**

Neuroimaging studies have proved that hippocampus relate to the deficient of memory in patients with post-traumatic stress disorder (PTSD). Many studies in healthy subjects also shown that insular cortex (IC) be involved in the declarative memory. This study was designed to investigate whether insular cortex is involved in declarative memory deficits in patients with PTSD.

**Methods:**

Twelve subjects with PTSD and 12 subjects without PTSD victims underwent functional magnetic resonance imaging and magnetic resonance imaging. All subjects performed encoding and retrieval memory tasks during the fMRI session. Voxel-based morphometry method was used to analyze gray-matter volume, and the Statistical Parametric Mapping (SPM2) was used to analyze activated brain areas when performing tasks.

**Results:**

Grey matter volume was significantly reduced bilaterally in the insular cortex of PTSD subjects than non-PTSD. PTSD group also had lower level of activation in insular cortex when performing word encoding and retrieval tasks than non-PTSD group.

**Conclusion:**

The study provides evidence on structural and function abnormalities of the insular cortex in patients with PTSD. Reduced grey-matter volume in insular cortex may be associated with declarative memory deficits in patients with PTSD.

## Background

The insular cortex (IC) is a region located in the centre of the cerebral hemisphere. It processes sensory input in all modalities: gustatory, olfactory, auditory, visual and somatosensory [1,2]. Although IC is considered primarily as a taste area and is involved in conditional taste aversion and taste recognition, some studies demonstrated the involvement of IC in face recognition, tactile recognition and working memory[3,4]. Results of two animal studies also suggest that the IC is involved in declarative memory. For instance, Bermudez-Rattoni reported that IC is involved in consolidation of memory, and the study by Miranda suggested that cholinergic transmission in the IC is necessary for the acquisition and consolidation of contextual memory[5,6].

Studies on PTSD suggest a specific association between the traumatic stress and changes in memory functions [7-9]. Patients with PTSD may suffer from long-term memory deficits. In Archibald and Tuddenham's follow-up study, many veterans of World War II still suffered from episodes of 'black-outs' or impairment of explicit memory[10]. Intrusive memories and impoverished memories are common complaints among patients with PTSD[7]. Intrusive memories are diagnostic symptoms in patients with PTSD; they were easily triggered by ordinary stimuli such as low-flying airplane or loud noise, or anything that relives any aspect of the traumatic event. These intrusive memories are accompanied by autonomic hyperarousal that may be experienced as reenactments of the original trauma (flashbacks)[11]. Impoverished memory includes deficits in declarative memory, fragmentation of memories, and trauma-related amnesia[7]. Declarative memory (explicit memory) refers to the ability to consciously remember and reproduce events and facts. Some studies demonstrated declarative memory deficits in PTSD [12-15].

IC may be involved in declarative memory deficits in patients with PTSD. Previous studies indicate that hippocampus plays an important role in the declarative memory deficits in PTSD, and most neuroimaging studies on patients with PTSD showed hippocampal atrophy[16]. Apart from hippocampal involvement, studies have shown involvement of other areas in memory processing. For instance, functional imaging studies on healthy subjects have shown that prefrontal cortex, medial temporal lobe (MTL) and cerebellum are active during the encoding process of episodic memory; while prefrontal cortex, anterior cingulate cortex (ACC), MTL, occipital lobe are active during the retrieval process of declarative memory [17-21]. Recent studies using fMRI (functional Magnetic resonance imaging) and PET (Positron Emission Tomography) have shown that IC is involved in higher cognitive functions. Activities that involve social interactions (competition and cooperation) are associated with increased activation in the anterior IC (BA13) [22]. When implementing tasks of declarative memory such as encoding and retrieving verbal or picture materials, the IC of normal subjects shows high level of activation [23-27]. In patients with schizophrenia, the activation of IC was lower than healthy subjects when they implemented the declarative memory task [28]. Taken together, these studies suggest that IC might be involved in declarative memory.

While results from animal studies and human neuroimaging studies support the involvement of IC in the declarative memory in healthy subjects, there is paucity of evidence on the relationship between IC and declarative memory deficit in patients with PTSD. Using structural and functional MRI, we aimed to examine the structural and functional differences of IC in surviving victims of a fire disaster with and without PTSD. Based on previous findings, we hypothesize that structural and functional changes in IC of patients with PTSD may be associated with deficits in declarative memory. To the best of our knowledge, our study is the first study to report on the relationship between the IC and declarative memory deficits in patients with PTSD.

## Methods

### Participants

Twelve patients (8 females and 4 males) with PTSD, and twelve subjects (8 females and 4 males) without PTSD, were recruited. All of them were recruited from 157 victims surviving a fire disaster occurred in November 2003 in Hunan province in China. After the PTSD screening and diagnostic program, 21 patients with PTSD were found and 15 patients consented to be recruited in this study, during the neuroimaging test, 3 patients dropped out. So the final subjects were 12 in the PTSD patients group.

We established PTSD diagnoses using the Structured Clinical Interview for DSM-IV[29] (First et al, 1995) and assessed the severity of PTSD using the Chinese version of the Distress Event Questionnaire (DEQ) [30,31]. The two groups did not differ significantly in age (34.56 years ± 4.91 [PTSD] and 33.25 years ± 5.27 [non-PTSD]; *t*[22] = -0.49, *p *= 0.68). The PTSD group had higher scores on DEQ than did the non-PTSD group (43.12 ± 5.61 [PTSD] and 12.58 ± 4.92 [non-PTSD]; *t*[22] = 4.46, *p *= 0.000). The presence of other psychiatric disorders was also assessed with the Structured Clinical Interview for DSM-IV (First et al 1995). No subject met the diagnostic criteria for major depression, schizophrenia, bipolar disorder, alcohol and substance abuse. Subjects were excluded if they had any clinical significant abnormality of a clinical laboratory test, a history of psychiatric illness or neurological dysfunction, a history of alcohol and/or drug abuse (DSM-IV criteria) within 6 months prior to the study, or claustrophobia. None of the participants were taking psychotropic drugs at the time of the study.

The following comorbid DSM-IV Diagnoses were found in the PTSD group: dysthymia (*n *= 2), specific phobia (*n *= 1), and generalized anxiety disorder (*n *= 3). None of the subjects in the non-PTSD group had current diagnoses. In both groups, no subject (with or without PTSD) had ever received psychiatric treatment for PTSD caused by the fire disaster. Moreover, none of the subjects had received any psychotropic treatment.

Verbal informed consent was obtained from each participant before participation because most victims didn't want to sign any paperwork. The Institutional Review Board of Central South University Xiangya Medical School approved this study in writing, and accepted the switch from written informed consent to the verbal informed consent.

### Tasks

Block design was used in the present study. Subjects were imaged during two functional runs while performing these encoding tasks. Each functional run lasted 300 sec and was comprised of 10 blocks, 5 of these were "task" blocks and 5 were "fixation" control blocks. During the fixation control blocks, a cross-hair (plus sign) was present on the screen for the duration of the block, and subjects were instructed to fixate the cross-hair. Task blocks (30 second duration) were interleaved with fixation blocks (30 second duration). 10 sets of Chinese words were presented during each task block (2000 msec stimulus duration, 1000 msec inter-stimulus interval). During the trial, subjects viewed a set of Chinese words (one pair of Chinese characters per trial) displayed simultaneously on a screen. One of the two characters in each pair was highlighted with a red arrow placed under the word. Subjects were instructed to remember words with a marker (target words) so that they would recognize it in the next task. Target words were randomly placed on the left or right screen. Subjects indicated their recognition of the target words using a keypad consisting of two horizontally arranged buttons, labeled as No. 1(left) and No. 2 (right). When the target word appeared on the left, subjects were instructed to press button No. 1.; vice-versa when the target word appeared on the right. The behavioral data (i.e., response times and percent of correct response) were recorded by the computer program.

Two seconds after the encoding scan, subjects were given an old-new recognition test. This trial consisted of 25 encoded words and 25 "new" words that were not presented during the encoding trial. There were also 5 "task" blocks and 5 "fixation" control blocks. The control block was presented in the same way as in the encoding trial. During the task block, one Chinese word was presented on the screen every time (2000 msec stimulus duration, 1000 msec inter-stimulus interval). 5 target words and 5 "new" words were presented in one task block. Each task block and control block lasted 30 seconds. Subjects indicated that an old word was presented by pressing button No. 1, or else, subjects pressed button No. 2,

### Imaging acquisition

The tasks were run on a PC laptop using E-Prime presentation software (Psychology Software Tools) and displayed to the subjects using a color LCD projector (Epson, ELP-7000). Stimuli on the screen were visible to the subject via a mirror (1.5 in × 3 in) positioned approximately 15 cm above the subject's eyes. Functional MRI data were collected with a 1.5-T whole-body scanner (General Electric Medical Systems Signa, Milwaukee, WI) with a standard head coil. Cushions were used to minimize head movement. Anatomic images were acquired using a high-resolution 3-D spoiled gradient recovery sequence (SPGR, slice thickness 1 mm, TR = 25 msec, TE = 6 msec, flip angle = 258, matrix = 256 × 128, FOV 24 × 24 cm). Functional data were acquired using a gradient-echo EPI pulse sequence (GRE-EPI, TR = 3 s, TE = 60 ms, matrix = 64 × 64, flip angle = 90°, FOV = 24 × 24 cm, slice thickness = 5 mm, skip between slices = 1.5 mm).

### fMRI and MRI Data Analyses

Data were analyzed with statistical parametric mapping (SPM2 software from the Wellcome Department of Cognitive Neurology, London, UK), running under Matlab 6.0 (Mathworks, Sherbon, MA).

### fMRI data

The fMRI data were realigned, spatially normalized to the standard brain space, and smoothed with an isotropic Gaussian kernel of 8 mm full width at half maximum (FWHM). Low-frequency noise and global changes in activity were further removed. For each participant, task-specific effects were estimated using a general linear model (modeled as a box-car function convolved with the canonical homodynamic response function). For random effects analysis, a contrast image between tasks and control was generated for each participant and used for intersubject comparisons.

#### 2.6 MRI data

Automated voxel-based morphometry (VBM) method was used in order to minimize operational biases when comparing the Neuroanatomical differences between patients with PTSD and the control subjects, [32,33]. VBM was used recently in structural MRI studies of various neuropsychiatric disorders [34]. This method was used by Yamasue (2003)[35] in another study on PTSD. The MRI data were first spatially normalized into the standard space of Talairach and Tournoux [36]. Second, normalized images were segmented into gray matter, white matter, cerebrospinal fluid, and skull scalp compartments by using an automated process. Third, the spatially normalized segments of the gray and white matters were smoothed with a 12-mm full-width, half-maximum isotropic Gaussian kernel to accommodate individual variability. Then, the partial-volume effect was used to create a spectrum of gray or white matter intensities by smoothing the data, Gray or white matter density is equivalent to the weighted average of the gray or white-matter voxel located in the volume defined by the smoothing kernel, and according to previous studies[33,34,37], the regional gray or white matter density can be considered to represent the local amount of gray or white matter.

### Statistical Analysis

For fMRI and MRI data, using an analysis of Two-sample t-test model running in SPM2, the significance level was set at corrected p < 0.05, performance between PTSD and controls was statistically compared. Furthermore, to rule out potential confounding factors that may affect fMRI and VBM findings, Simple Regression Analysis (in SPM 2) was also performed taking into account symptom measures and demographic data (age, gender, and years of education.) in victims with and without PTSD separately. Statistical significance was defined at corrected P < 0.05.

## Results

### Performance

Performance data are summarized in Additional file 1. Behavioral measures of accuracy (retrieval task) and reaction time (encoding and retrieval tasks) were acquired for all subjects. In encoding task, there were no differences in reaction time between PTSD patients and comparison subjects (Z = 0.52, p > 0.05) (see Additional file 1). In the retrieval task, patients tend to have lower accuracy in recognizing targets and longer response time. For instance, there were significant differences in reaction time (Z = 4.21, P < 0.001) and in response bias (X = 16.98, P < 0.001).

### Imaging results

#### Encoding task

Additional file 2 presents local maxima for encoding task. As in previous studies [38-40], the comparison subjects had extensive frontal activation, including bilateral Broca's area (Brodmann's area 6), right frontal pole (Brodmann's area 10), left dorsolateral prefrontal cortex (Brodmann's area 46) and bilateral inferior frontal Gyrus (Brodmann's area 47). The comparison subjects also had activation in the right cingulate Gyrus (Brodmann's area 31), bilateral anterior cingulate (Brodmann's area 24, 25), bilateral parahippocampal (Brodmann's area 30), left hippocampus and bilateral insular cortex (Brodmann's area 13). Like the comparison subjects, the patients activated the bilateral Broca's area (Brodmann's area 6), left dorsolateral cortex (Brodmann's area 46), left hippocampus, left insular (Brodmann's area 13) and bilateral parahippocampal (Brodmann's area 34, 35). However, the patients did not activate the right cingulate Gyrus (Brodmann's area 31), bilateral anterior cingulate (Brodmann's area 24, 25), and the right insular cortex (Brodmann's area 13).

Between-group contrasts (Additional file 2, Figure 1) revealed that the comparison subjects had greater frontal activation in the right superior frontal Gyrus (Brodmann's area 8), bilateral middle frontal (Brodmann's area 6, 8) and bilateral inferior frontal Gyrus (Brodmann's area9, 45, 46). The comparison subjects also had great activation in the left hippocampus, bilateral parahippocampal (Brodmann's area 30), right cingulate Gyrus (Brodmann's area 31), bilateral anterior cingulate Gyrus (Brodmann's area 24, 25) and left insular cortex (Brodmann's area 13)

**Figure 1:**
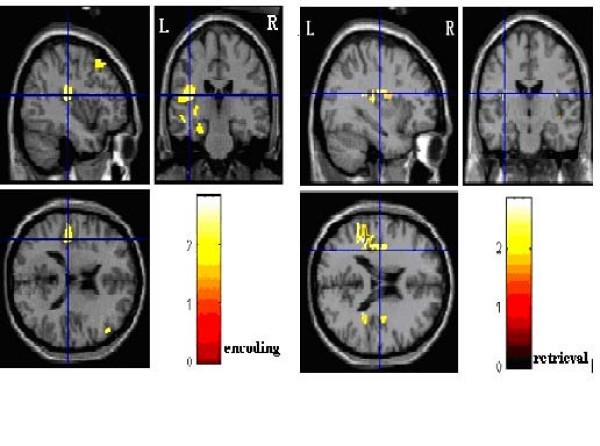
**Insular cortex activation showed great difference between PTSD and controls**. In encoding task, activation of left insular in controls was greater than that in patients with PTSD (left). In retrieval task, activation of bilateral insulars in comparison subjects was greater than that in patients with PTSD (right).

#### Retrieval task

Additional file 3 presents local maxima for retrieval task. The comparison subjects had extensive prefrontal cortex activation, including activation in the right superior pole (Brodmann's area 10), and bilateral Broca's area (Brodmann's area 6, 9). The comparison subjects also showed bilateral activation of cingulate Gyrus (Brodmann's area 24, 31), left activation of parahippocampal Gyrus (Brodmann's area 36), bilateral activation of hippocampus and ICs. Patients with PTSD also had activation in the right superior frontal Gyrus (Brodmann's area 6), bilateral activation of middle frontal Gyrus (Brodmann's area 9, 10), left inferior frontal Gyrus (Brodmann's area 44) and right parahippocampal Gyrus (Brodmann's area 30). However, in the patients group, there were no significant activations in bilateral cingulate Gyrus, bilateral hippocampus and bilateral ICs.

Between-group contrasts (Additional file 3, Figure 2) showed greater activation in frontal regions (Brodmann's area 6, 10, 11, 38), limbic cortex (bilateral hippocampus, bilateral anterior cingulate cortex), and bilateral ICs (Brodmann's area 13) of the comparison subjects.

**Figure 2:**
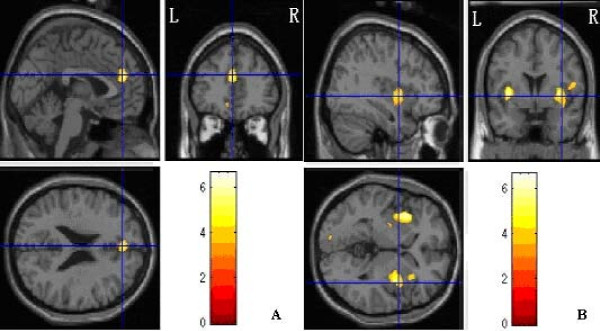
**Regional differences between PTSD group and control group**. A showed that left Medial Frontal Gyrus (Brodmann's area 9) with significantly reduced gray-matter densities in PTSD group compared with controls. B showed that bilateral insulars (Brodmann's area 13) with significantly reduced gray-matter densities in PTSD compared with controls. Images were rendered onto orthogonal slices of the normal template magnetic resonance images.

#### Morphological comparison

MRI images were analyzed by VBM method so as to compare whether there was morphological difference between PTSD and controls or not. Results revealed that regions with less gray-matter density in PTSD group compared with control group included left Medial Frontal Gyrus (Brodmann's 9) {peak coordinate (Talairach) [*x *= *-1*, *y *= *41*, *z *= *21*], *T *score = 5.05}, and bilateral ICs (Brodmann's 13) {the left IC, peak coordinate (Talairach) [*x *= *-36*, *y *= *2*, *z *= *0*], *T *score = 4.64; the right IC, peak coordinate (Talairach) [*x *= *34*, *y *= 4, *z *= *6*], *T *score = 4.44;}. The intensities in other gray-matter regions and any of the white-matter regions did not show any significantly differences between two groups. These results indicated left Medical Frontal Gyrus and bilateral volume had significantly reduction in patients with PTSD than in the controls.

## Discussion

The IC is located along the rhinal sulcus, rostra to the peripheral cortex. It is involved in the processing of visceral sensory, visceral motor, vestibular, attention, pain, emotion, verbal, motor information, inputs related to music and eating, in addition to gustatory, olfactory, visual, auditory, and tactile data. Recent neuroimaging data, including voxel based morphometry, PET and fMRI, revealed that IC was involved in various neuropsychiatric diseases such as mood disorders, panic disorders, PTSD, obsessive-compulsive disorders, eating disorders, and schizophrenia. Investigations of functions and connections of the IC suggest that sensory information including gustatory, olfactory, visual, auditory, and tactile inputs converge on IC, and that these multimodal sensory information may be integrated there.

The goal of the current study is to examine whether the IC (Brodmann's area 13) may be involved in declarative memory deficits in PTSD. Based on results of fMRI and MRI analysis, the IC may be involved in declarative memory deficits in PTSD. In encoding and retrieval tasks (tasks of declarative memory), the activation of the IC in patients with PTSD was lower than that in comparison subjects. Furthermore, gray-matter volume of bilateral ICs in patients with PTSD had greatly decreased.

Connections between the IC and other parts of cortex were extensive [41,42]. These include connections with the prefrontal cortex (orbital cortex, medical prefrontal cortex), the limbic system (anterior cingulate cortex, amygdala), and the temporal pole had been documented [41]. IC has also been implicated as a visceral sensory area, visceral motor area, motor association area, area, and language area. It thus plays important roles in somatosensory integration, pain perception [41,43], and the experience of some emotional states, especially disgust [44-46]. Until now, increasing evidence has been showing the involvement of IC in emotional processing[46]. Human subjects reported fearful emotion when their IC cortex was stimulated by electricity[47], Anterior IC of patients with phobia were activated when their symptoms were provoked [48]. Activation of IC was also found in processing taste and recalling negative emotion (such as sadness, fear, disgust), [49-53].

Recent studies indicated that the IC was involved in cognitive processing. IC was involved in performing cognitively demanding emotional tasks [54]. In a meta-analysis of 43 PET and 12 fMRI activation studies that used the emotional activation paradigm, Phan suggested that anterior cingulate cortex and the IC were involved in emotional induction with cognitive demand[54]. Reiman found that emotional recall, but not emotional film viewing, engaged the IC[55]. When implementing two cognitive tasks (competition and cooperation), the anterior IC (Brodmann's area 13) activation increased [22]. The IC was activated when processing the task of suppressing all conscious thoughts[56]. Study of Chee indicated the left IC was a marker for language attainment in bilinguals[57]. A magnetic resonance imaging found that hippocampus, parietal cortex, and the IC had significantly more atrophy in patients with early Alzheimer's disease than in healthy controls[58]. Their data suggest that the IC may be involved early in Alzheimer's disease and that atrophy of the IC may contribute to the cognitive deficits typical of early Alzheimer's disease.

Moreover, the IC is involved in declarative memory. Two fMRI studies found BOLD signal of bilateral ICs increased when implementing the task of word recognition,[23,24]. Also, a fMRI study by Opitz (2000) found that bilateral ICs of normal subjects were activated during the word encoding and retrieval task[27]. Similar results were also reported in other neuroimaging studies on memory. The activation of IC was great when encoding and retrieving materials in episodic memory, and encoding memory tasks with pictures material [25,26].

The hippocampus is plays key role in declarative memory [59-63]. Several structural MRI studies have found smaller hippocampus in patients with PTSD. In a recent positron emission tomography (PET) and MRI study in women with PTSD related to childhood sexual abuse, Bremner (2003) found decreased hippocampal blood flow in patients with PTSD compared to controls during paragraph encoding. In another paradigm, women with PTSD showed greater decreases in blood flow in frontal cortex and left hippocampus, and increases in visual association and motor cortex during recall of emotionally valenced word pairs[64]. Another PET study of word-stem completion also revealed an abnormal rCBF response in the hippocampus in firefighters with PTSD[52]. Squire (1996) considered that the hippocampal formation consists of two components: the hippocampus and the entorhinal cortex[65,66]. The entorhinal cortex is the major source of cortical projections to the hippocampus region. It also receives other direct inputs from the olfactory bulb, orbital frontal cortex, the IC cortex, cingulate cortex and superior temporal Gyrus. That means the IC may be involved in declarative memory via hippocampus. In our study, the PTSD group with deficits in declarative memory had less activation in orbital frontal cortex (Brodmann's area 9, 10), hippocampus, cingulate and the IC than controls (Additional file 1, Additional file 2), when implementing encoding and retrieval tasks. These results are consistent with recent findings from study of Regland[28]in which patients with schizophrenia who had significant cognitive deficits showed less bilateral ICs activation (the left IC when encoding, the right IC with recognition, Brodmann's area 13) than healthy comparison subjects when processing word encoding and retrieval tasks.

Our study also suggests that the IC may be involved when patients experience symptoms of PTSD. Critchley (2001) proposed that the IC is involved in representing states of awareness related to external threat as well as in representing internal states of arousal[67]. Cortical regions involved in subjective awareness and states of bodily arousal during fear conditioning also include the anterior IC and adjacent orbit frontal cortices[68]. Autonomic hyperarousal symptom is one of the core symptoms of PTSD. The IC was shown to play an important role in suppressing conscious thoughts[56], which means that deficits of IC may be responsible for the recurring and intrusive nature of traumatic memories.

There are several further limitations of the present study. One of the limitations is that the sample size of the groups was relatively small; this did not allow application of alternative statistical models. Investigations with larger sample sizes are currently in progress. Another limitation is that the connection between IC and the declarative memory deficits in PTSD need more strong evidences not just from the function activation analysis but also further functional connectivity analyses.

## Conclusion

To conclude, VBM analysis in our study showed bilateral reduction in IC gray-matter volume in subjects with PTSD. Furthermore, the fMRI data showed that there was less IC activation in people with PTSD than the comparison subjects when performing word encoding and retrieval tasks. These findings suggest that IC may be involved in declarative memory deficits in PTSD, which may be implicated in the symptom generation of PTSD.

## Competing interests

The authors declare that they have no competing interests.

## Authors' contributions

SC carried out the study, participated in the sequence alignment and drafted the manuscript. LL participated in the design of the study, conceived of the study. BX and JL participated in the sequence alignment. All authors read and approved the final manuscript.

## Pre-publication history

The pre-publication history for this paper can be accessed here:



## Supplementary Material

Additional file 1 **Table 1**. Performance during Encoding and Retrieval Tasks for Patients with PTSD and Comparison subjects. Click here for file

Additional file 2 **Table 2**. Local Maxima of Blood-Oxygen-Level-Dependent fMRI Signal Change during Encoding in Comparison Subjects and Patients with PTSD. Notes in Table 2 and Table 3. Bold means interesting areas. ^a ^Peak activation in a cluster of at least ten voxel in which the difference in signal change exceeded an extent and threshold corrected p value of 0.05. ^b ^Coordinates from the stereotaxic atlas of Talairach and Tournoux. Click here for file

Additional file 3 **Table 3**. Local Maxima of Blood-Oxygen-Level-Dependent fMRI Signal Change during Retrieval in Healthy Comparison Subjects and Patients with PTSD. Notes in Table 2 and Table 3. Bold means interesting areas. ^a ^Peak activation in a cluster of at least ten voxel in which the difference in signal change exceeded an extent and threshold corrected p value of 0.05. ^b ^Coordinates from the stereotaxic atlas of Talairach and Tournoux. Click here for file
