# Supplementary material for: Insular cortex involvement in declarative memory deficits in patients with post-traumatic stress disorder
Source: BMC Psychiatry. 2009 Jun 18;9:39. doi: 10.1186/1471-244X-9-39 (PMC2704184; doi:10.1186/1471-244X-9-39)
Supplement: Additional file 1 — Table 1. Performance during Encoding and Retrieval Tasks for Patients with PTSD and Comparison subjects. [file 1471-244X-9-39-S1.doc]

TABLE 1.

| Patients(N=12) Comparison subjects(N=12)  Task and Performance Measure Mean SD Mean SD p |
| --- |
| Encoding  Reaction time (msec) 978.93 137.80 957.70 130.15 >0.05  Retrieval  Reaction time (msec) 1182.93 165.53 938.40 125.41 <0.001  Accuracy(percent) 74.19 4.56 84.00 2.00 <0.001 |
