# Supplementary material for: Insular cortex involvement in declarative memory deficits in patients with post-traumatic stress disorder
Source: BMC Psychiatry. 2009 Jun 18;9:39. doi: 10.1186/1471-244X-9-39 (PMC2704184; doi:10.1186/1471-244X-9-39)
Supplement: Additional file 2 — Table 2. Local Maxima of Blood-Oxygen-Level-Dependent fMRI Signal Change during Encoding in Comparison Subjects and Patients with PTSD. Notes in Table 2 and Table 3. Bold means interesting areas. a Peak activation in a cluster of at least ten voxel in which the difference in signal change exceeded an extent and threshold corrected p value of 0.05. b Coordinates from the stereotaxic atlas of Talairach and Tournoux. [file 1471-244X-9-39-S2.doc]

Table 2

| Group and Region Estimated Brodmann’s Area zScore^a^ Coordinates^b^  X Y Z |
| --- |
| **Comparison subjects (n=12)**  Left superior frontal Gyrus 6 4.19 -61 6 30  Right superior frontal Gyrus 6 3.76 53 -4 30  Right middle frontal Gyrus 10 5.38 32 54 -8  Left middle frontal Gyrus 46 4.78 -55 30 19  Left inferior frontal Gyrus 47 4.22 -53 35 -2  Right inferior frontal Gyrus 47 3.06 22 13 -21  Right anterior cingulate Gyrus 25 5.57 6 2 -3  Left anterior cingulate Gyrus 24 5.77 -6 -1 24  Right cingulate Gyrus 31 4.81 4 -47 41  **Left hippocampus 9.00 -28 -29 -4**  **Right parahippocampal 30 4.05 12 -41 4**  **Left parahippocampal 30 4.73 -18 -46 4**  **Left insular cortex 13 4.37 -44 14 1**  **Right insular cortex 13 3.56 44 8 0**  **PTSD patients(n=12)**  Right superior frontal Gyrus 6 1.87 6 16 58  Right superior frontal Gyrus 10 2.34 30 50 25  Right middle frontal Gyrus 6 2.38 38 12 47  9 2.55 53 23 36  46 2.72 48 27 32  Left middle Frontal Gyrus 46 1.99 -50 25 26  9 3.04 -42 29 35  **Left hippocampus 2.20 -28 -31 -3**  **Left parahippocampal 35 2.07 -24 -28 -12**  **Right parahippocampal 36 1.88 24 -33 -10**  **Left insular cortex 13 1.99 -40 10 3**  **Comparison subjects > PTSD patients**  Right superior frontal Gyrus 8 1.77 22 16 45  Right middle frontal Gyrus 6 2.44 36 12 47  Left inferior frontal Gyrus 9 1.86 -51 17 23  Right inferior frontal Gyrus 45 1.89 53 37 0  Left anterior cingulate Gyrus 24 1.77 –6 -1 24  Right anterior cingulate Gyrus 25 1.84 2 0 -3  Right Cingulate Gyrus 31 1.81 4 -47 41  **Left hippocampus 2.19 -20 -29 -4**  **Right parahippocampal 30 2.05 12 -41 4**  **Left Parahippocampal 30 1.73 -18 -46 4**  **Left Insular 13 2.17 -40 -22 18** |
