# Supplementary material for: Insular cortex involvement in declarative memory deficits in patients with post-traumatic stress disorder
Source: BMC Psychiatry. 2009 Jun 18;9:39. doi: 10.1186/1471-244X-9-39 (PMC2704184; doi:10.1186/1471-244X-9-39)
Supplement: Additional file 3 — Table 3. Local Maxima of Blood-Oxygen-Level-Dependent fMRI Signal Change during Retrieval in Healthy Comparison Subjects and Patients with PTSD. Notes in Table 2 and Table 3. Bold means interesting areas. a Peak activation in a cluster of at least ten voxel in which the difference in signal change exceeded an extent and threshold corrected p value of 0.05. b Coordinates from the stereotaxic atlas of Talairach and Tournoux. [file 1471-244X-9-39-S3.doc]

Table 3

| Group and Region Estimated Brodmann’s Area zScore^a^ Coordinates^b^  X Y Z |
| --- |
| Comparison subjects (N=12)  Right superior frontal Gyrus 10 5.77 26 63 13  Left superior frontal Gyrus 9 6.47 -46 17 -9  Right superior frontal Gyrus 6 6.34 8 12 55  Left anterior cingulate Gyrus 24 3.65 -14 -10 35  Right posterior cingulate Gyrus 31 3.43 22 -42 22  Left parahippocampal Gyrus 36 3.22 -40 -32 -12  **Right hippocampus 2.96 32 -18 -19**  **Left hippocampus 3.15 -30 -22 -17**  **Left insular cortex 13 4.63 -42 -34 18**  **Right insular cortex 13 4.42 42 -32 15**  **Patients (N=12)**  Right superior frontal Gyrus 6 3.17 10 11 66  Left Middle Frontal Gyrus 9 4.81 -12 42 18  Right Middle Frontal Gyrus 10 3.69 8 55 -8  Left Inferior Frontal Gyrus 44 3.28 -59 16 16  Right Parahippocampal Gyrus 30 2.98 16 -33 -6  **Comparison Subjects > Patients**  Right Superior Frontal Gyrus 6 3.34 8 12 55  Left Middle Frontal Gyrus 6 3.33 -38 14 53  Right Middle Frontal Gyrus 10 3.29 24 63 6  Left Anterior Cingulate Gyrus 24 2.99 -14 -10 35  Right Posterior Cingulate Gyrus 31 3.43 22 -42 22  Left Parahippocampal Gyrus 36 3.01 -40 -32 -12  **Left Hippocampus 3.85 -30 -22 -17**  **Right hippocampus 3.70 32 -18 -19**  **Left Insular 13 3.51 -42 -34 18**  **Right Insular 13 3.62 42 -32 15** |
